# Characterization, antifungal potential, cytocompatibility, and regenerative potential of mucoadhesive gel containing antifungal‐β‐cyclodextrin inclusion complexes

**DOI:** 10.1111/jopr.70135

**Published:** 2026-04-11

**Authors:** Carolina Yoshi Campos Sugio, Amanda Aparecida Maia Neves Garcia, Diana Gabriela Soares, Fernanda Balestrero Cassiano, Marcela Alves de Mattos, Simone Soares, Priscileila Colerato Ferrari, Maria Helena Fernandes, Vanessa Migliorini Urban, Karin Hermana Neppelenbroek

**Affiliations:** ^1^ Department of Prosthodontics and Periodontics Bauru School of Dentistry University of São Paulo (USP) Bauru São Paulo Brazil; ^2^ Department of Removable Prosthodontics and Dental Materials Dental School, Federal University of Uberlândia (UFU) Uberlândia Minas Gerais Brazil; ^3^ Department of Operative Dentistry Endodontics and Dental Materials Bauru School of Dentistry University of São Paulo (USP) Bauru São Paulo Brazil; ^4^ Department of Dentistry State University of Ponta Grossa (UEPG) Ponta Grossa Paraná Brazil; ^5^ Department of Pharmaceutical Sciences State University of Ponta Grossa (UEPG) Ponta Grossa Paraná Brazil; ^6^ BoneLab‐Laboratory for Bone Metabolism and Regeneration Faculty of Dental Medicine University of Porto (UP), LAQV, REQUIMTE, University of Porto Porto Portugal

**Keywords:** *Candida albicans*, candidiasis, chlorhexidine, cyclodextrins, drug delivery systems, nystatin, oral

## Abstract

**Purpose:**

This study aimed to characterize a mucoadhesive gel containing nystatin (NYS) or chlorhexidine (CHX), complexed or not with β‐cyclodextrin (βCD), and evaluate its rheological and mucoadhesive properties, antifungal efficacy, cytocompatibility, and collagen synthesis potential.

**Materials and Methods:**

Mucoadhesive formulations were produced using chitosan and hydroxyethylcellulose, incorporating NYS or CHX at 32 mg/g, or complexed with βCD (NYS:βCD, equivalent to 16.1 mg/g of NYS, and CHX:βCD, equivalent to 4.8 mg/g of CHX), and compared to a pure gel (GEL) and a commercially available 2% miconazole gel (DK). The formulations were evaluated for their rheological and mucoadhesive properties; antifungal activity against *Candida albicans* by halo inhibition, XTT metabolic assay, minimum inhibitory concentration (MIC) and minimum fungicidal concentration (MFC); cytocompatibility in an organotypic oral mucosa model by LIVE/DEAD and Alamar Blue metabolic assay; and collagen synthesis potential by fluorometric quantification. All microbiological and cytocompatibility tests were performed using mucoadhesive leachates. Statistical analysis was performed (*α* = 0.05).

**Results:**

Elastic behavior was predominant in the formulations, and greater mucoadhesiveness was observed in the complexed groups (*p* < 0.05). Limited antifungal activity was found in the GEL and DK groups, while the NYS, NYS:βCD, CHX, and CHX:βCD formulations exhibited significantly superior antifungal efficacy (*p* < 0.05). While formulations containing CHX significantly reduced cellular metabolic activity, those containing NYS demonstrated better cytocompatibility and increased collagen production.

**Conclusion:**

The mucoadhesive gel containing NYS:βCD optimized both antifungal activity without compromising its rheological, mucoadhesive properties, and cytocompatibility at lower therapeutic doses compared to noncomplexed NYS. These findings suggest significant advantages for the treatment of oral candidiasis.

Oral candidiasis, mainly caused by *Candida albicans*,^1^ is a common fungal infection that affects the mucosal surfaces of the oral cavity and is very frequent in immunocompromised individuals^2^ and denture wearers.^3^ Topical antifungal agents, especially miconazole 2% gel^4^ and nystatin (NYS) oral suspension,^5^, ^6^ are considered the first‐line treatment for uncomplicated cases of oral candidiasis. Although effective in improving the signs and symptoms of infection, topical antifungal drugs have problems related to maintaining their efficacy after the end of treatment, which has been related to the short retention time in the mucosa and, consequently, the low bioavailability of these drugs due to salivary flow, tongue movements, and swallowing.^7^

Mucoadhesive formulations carrying antifungal agents have emerged as a promising alternative for the treatment of oral candidiasis, as they can prolong the drug residence time at the site of infection by adhering to the mucosal surface, thus enhancing therapeutic results.^8^, ^9^ Mucoadhesive systems have also been shown to offer the advantage of providing controlled and prolonged drug release, maintaining the therapeutic concentration through optimization of bioavailability, which allows a reduction in the frequency of daily application.^9^

NYS oral suspension is one of the most commonly prescribed medications for the treatment of oral candidiasis, primarily due to its lower potential for adverse effects, since the formulation is not absorbed by the gastrointestinal tract.^6^ However, in addition to the short retention time at the infection site, the effectiveness of NYS therapy is compromised by its low solubility and limited absorption by the mucosa.^10^ Chlorhexidine (CHX) is a broad‐spectrum antimicrobial agent frequently used in the treatment of oral infections, including candidiasis.^5^ Although effective, CHX is also a poorly soluble drug and has a cytotoxic profile that can damage oral tissues at higher concentrations.^11^, ^12^ Mucoadhesive formulations containing antifungals can increase the retention time in the mucosa, improving their efficacy, as well as allowing for lower doses, thus reducing their toxicity.^13^

Inclusion complexes are molecular assemblies in which a guest molecule, such as a drug, is encapsulated within the hydrophobic cavity of a host molecule, such as β‐cyclodextrin (βCD), without the formation of covalent bonds. The development of inclusion complexes has been suggested as a strategy to improve the solubility, stability, and bioavailability of poorly water‐soluble drugs, such as NYS^10^ and CHX.^14^ These complexes can encapsulate antifungal agents, offering an additional benefit in drug delivery systems.^15^ The combination of mucoadhesion and inclusion complex formation can optimize the therapeutic effect of antifungal drugs, addressing both the challenges of effective administration and the limitations of conventional treatment approaches. When incorporated into mucoadhesive gels, NYS and CHX complexed with βCD demonstrated greater release compared to the same drugs noncomplexed, corroborating the hypothesis that the clinical benefit stems from increased solubilization and retention of the drug in the mucosa.^13^

In the available literature, only investigations on mucoadhesive systems containing antifungal drugs evaluating the cytotoxicity of the formulations in preliminary analyses of cell monolayers were found.^16^, ^17^, ^18^, ^19^, ^20^ The main novelty of this study was the use of an organotypic oral mucosa model, which more accurately mimics the structural and functional characteristics of human oral tissues. This approach allowed for the investigation of cytocompatibility and collagen synthesis potential in a highly relevant biological context, which is essential to ensure the safety and therapeutic potential of new formulations for oral candidiasis prior to clinical trials in humans. Therefore, the aim of this study was to evaluate mucoadhesive formulations containing NYS and CHX complexed with βCD regarding rheological properties, mucoadhesive performance, in vitro antifungal activity, cytocompatibility in an organotypic model of the oral mucosa, and the ability to promote collagen production.

## MATERIALS AND METHODS

### Preparation of mucoadhesive formulation

The mucoadhesive gel formulation was prepared according to a previously developed methodology.^13^ Briefly, a 2% (w/v) chitosan (Low molecular weight 50,000–190,000; DA 75%–85% deacetylated; Sigma‐Aldrich Inc.) dispersion and a 6% (w/v) hydroxyethyl cellulose (HEC; Natrosol®, Ashland Inc.) solution were prepared using 1% (v/v) glacial acetic acid and distilled water, respectively. Preservatives were added to the dispersion, including methylparaben (0.15% w/w), sodium metabisulfite (0.1% w/w), and EDTA (0.1% w/w). The chitosan and HEC solutions were then mixed in a 1:3 weight ratio and homogenized at room temperature.

Noncomplexed NYS and CHX were incorporated into the mucoadhesive formulations at concentrations previously established as effective against *C. albicans* biofilms in polymer‐based systems,^20^, ^21^ without negatively affecting the physical–mechanical properties of the matrices.^22^, ^23^, ^24^ For the βCD‐complexed drugs, the concentrations were determined according to the physicochemical characterization and antifungal activity of the NYS:βCD10 and CHX:βCD14 inclusion complexes. These complexes were shown to form stable associations in molar ratios of 1:1 (NYS) and 1:2 (CHX), as reported in the original characterization studies.^10^, ^14^ These molar ratios guided the concentrations evaluated in the formulations tested in this investigation. Thus, the following study groups were established:

- GEL—unloaded mucoadhesive gel;
- DK—2% miconazole gel (Daktarin® Janssen); gel containing 20 mg/g miconazole;
- NYS—noncomplexed mucoadhesive gel containing 32 mg/g NYS;
- NYS:βCD—complexed mucoadhesive gel containing 36 mg/g inclusion complex NYS:βCD, equivalent to 16.1 mg/g NYS;
- CHX—noncomplexed mucoadhesive gel containing 32 mg/g CHX;
- CHX:βCD—complexed mucoadhesive gel containing 26 mg/g inclusion complex CHX:βCD, equivalent to 4.8 mg/g CHX.

### Rheological analysis

Rheological tests were performed in oscillatory mode, using a 40 mm cone‐plate geometry with a 0.4 mm gap. One gram of the mucoadhesive gel was placed on the lower plate of the oscillatory rheometer (Discovery Hybrid Rheometer‐2, TA Instruments), allowing for minimal sample shear. A 30‐s rest period was applied before each measurement. A frequency range of 0.1–100 Hz and an amplitude of 1% was used to analyze each sample at 37°C. The elastic modulus (*G*′), viscous modulus (*G*″), and loss tangent (tan(δ)) were determined.^25^

### Mucoadhesive properties

The mucoadhesive properties (*n* = 3) of the formulations were evaluated using a texture analyzer (Texturometer TX‐700, Lamy) in an ex vivo model with fragments of porcine buccal mucosa (CEUA: 0193028; UEPG Protocol: 20.000011339‐8). The samples were thawed (−80°C), washed with 0.9% saline, molded into rounds, and fixed to a 36 mm probe with cyanoacrylate glue (SuperBonder®, Loctite).

One gram of each mucoadhesive gel was placed in a Petri dish preheated to 37°C and fixed to the equipment base. The test applied 0.5 N compression for 120 s, followed by tension at 1 mm/s until the mucosa detached. Force–distance graphs were generated to determine adhesiveness (N), work of adhesion (N.s), which represents the energy required for detachment, and debonding distance (mm).^26^, ^27^

### Preparation of stock solution

For the development of the antifungal potential and cytotoxicity assessments, leachates from the mucoadhesive formulations were used. The formulations were incubated in a medium at a ratio of 1:3 g/mL at 37°C for 4 h.^13^, ^28^ The stock solution was obtained by spontaneous drug release in RPMI‐1640 medium buffered with 3‐(N‐morpholino)propanesulfonic acid (MOPS; Sigma‐Aldrich Inc.) for microbiological assays and in Dulbecco's modified Eagle medium (DMEM; Gibco/Thermo Fisher Scientific Inc.) for cell culture experiments.

### Antifungal potential

The *C. albicans* SC5314 strain was thawed and cultured on Sabouraud dextrose agar (SDA; Difco Laboratories Inc.) at 37°C for 24 h. The colonies were suspended in sterile water, and the cell density was adjusted to the 0.5 McFarland standard using a spectrophotometer (Synergy Mx, Biotek®) at 530 nm. The inoculum was then prepared by diluting the suspension with RPMI 1640 broth medium, according to the protocol M27‐A3.^29^

#### Inhibition halo

To evaluate the inhibitory potential of mucoadhesive gels, SDA plates were divided into four sections and inoculated with 50 µL of standardized *C. albicans* inoculum. After drying the surface for 2 min, 5‐mm‐diameter wells were aseptically prepared in the center of each quadrant of the Petri dishes using a sterile pipette tip. Subsequently, 35 µL of the leachates were carefully dispensed into each well.^30^, ^31^ The plates were incubated at 37°C for 24 h, and the inhibition zone was measured in three directions using a digital caliper. The halo was defined as the area with no visible colony growth under good lighting. The test was performed in quadruplicate on at least three separate occasions.

#### Reduction of tetrazolium salts—XTT

Mature biofilm formation was induced in a 96‐well plate at 37°C for 48 h. The biofilm was then centrifuged and washed twice with PBS. An aliquot of 200 µL of the mucoadhesive leachates from each experimental group was added to the wells, and the plate was incubated at 37°C for 24 h. After incubation, it was centrifuged again, washed with PBS, and 200 µL of XTT solution was added to each well, followed by incubation at 37°C for 3 h. Absorbance was measured at 450 nm using a spectrophotometer to assess metabolic activity based on mitochondrial function.^32^ The experiment was conducted in triplicate on three independent occasions.

#### Determination of minimum inhibitory concentrations

The minimum inhibitory concentration (MIC) was determined according to CLSI M27‐A5 (2017) guidelines.^33^ Serial dilutions of mucoadhesive leachates were prepared in a 96‐well plate using RPMI‐1640 medium. A 100 µL aliquot of a standardized *C. albicans* SC5314 inoculum was added to each well. The plate was incubated at 37°C for 24 h. The MIC was defined as the lowest concentration of the leachate that inhibited 90% of fungal growth (MIC_90_), assessed by measuring the absorbance at 550 nm using a spectrophotometer. The experiment was conducted in triplicate on three independent occasions.

#### Determination of minimum fungicidal concentrations

After determining the MIC, the contents of each well were seeded in duplicate using the microdrop technique. A 10 µL aliquot was transferred onto an SDA plate and incubated at 37°C for 24 h. After incubation, *C. albicans* colonies were counted to determine the colony forming unit per milliliter (CFU/mL) values. Minimum fungicidal concentrations (MFCs) were defined as the lowest concentrations of noncomplexed or complexed antifungals in the mucoadhesive system that completely inhibited fungal growth.^34^

### Cytocompatibility tests in an organotypic model of the oral mucosa

The cytocompatibility of the mucoadhesive formulations at the determined MICs_90_ and MFCs was evaluated using an in vitro model. These included spontaneously immortalized normal oral keratinocytes (NOK‐Si; RRID:CVCL_BW57) and a three‐dimensional (3D) culture of human gingival fibroblasts (HGFs; CAAE: 26153119.6.0000.5417), derived from healthy gingival tissue via enzymatic digestion with collagenase type I (Collagenase, Type I, Worthington Biochemical Corp.). HGFs were cultured in a 3D collagen matrix by dissolving type I collagen (3.89 mg/mL) in 10× culture medium at 4°C, followed by pH adjustment with 5 M NaOH. A total of 1 × 10^5^ HGFs in 10 µL was incorporated into the collagen solution and incubated at 37°C with 5% CO_2_ for 30 min to allow gelation. To simulate an organotypic oral mucosa model, a monolayer of NOK‐Si cells (5 × 10^4^ cells/membrane) was formed at the bottom of the plate, and 3D cultures of HGF (2 × 10^5^ cells/200 µL) were placed in the upper compartment of a 24‐well transwell system (Transwell Permeable Supports, 0.4 µm Pore, Corning), following the protocol adapted from Alamo et al.^32^ After 24 h of incubation, 1 mL of mucoadhesive leachates at their MICs_90_ and MFCs concentrations was added to each well, and the plates were incubated for an additional 24 h. The Gel and Dk groups were included for comparison and statistical analysis. The negative control was cells incubated in pure culture medium.

#### Cell viability (LIVE/DEAD assay)

To evaluate cell viability (LIVE/DEAD assay) at 24 and 72 h (*n* = 2), cells and 3D cultures were washed with PBS and incubated for 15 min at room temperature in culture medium containing 4 mM Calcein AM (green fluorescence = viable cells) and 2 mM ethidium homodimer‐1 (red fluorescence = dead cells). After incubation, transwells and 3D cultures were washed with PBS, spotted on glass slides, and analyzed for viable and nonviable cells using a fluorescence microscope (FLoid Cell Imaging Station, Applied Biosystems) at a magnification of ×20.^32^

#### Alamar blue assay

Cell metabolism was assessed quantitatively using the Alamar Blue assay following 24 and 72 h exposure (*n* = 6) of cells to mucoadhesive leachates. The 3D cultures of HGF and NOK‐Si keratinocytes were incubated for 4 h at 37°C and 5% CO_2_ in DMEM without FBS, supplemented with AlamarBlue cell metabolism reagent (10:1) (Life Technologies). After incubation, the supernatant was transferred to a 96‐well plate (CELLSTAR®; Greiner Bio‐One), and fluorescence was measured at 540 nm excitation and 590 nm emission. The fluorescence value from the control group at 24 h was used as a baseline (100%) to calculate cell proliferation.^32^

### Collagen synthesis potential

The collagen content in the supernatants of HGF cells (*n* = 4) was assessed after 24 and 72 h of exposure to mucoadhesive leachates. HGF cells (≈ 2 × 10^4^ cells/mL) were incubated with the leachates, lysed with 1% Triton, centrifuged, and diluted in 100 µL ultrapure water (based on the standard curve of the experiment). Aliquots (20 µL) of each sample were transferred to a 96‐well plate, and collagen content was determined according to the manufacturer's instructions (MAK322, Collagen Assay Kit, Sigma‐Aldrich Inc.). Results were expressed as a percentage, with the negative control (cells incubated with pure culture medium) defined as 100%.^32^

### Statistical analysis

Statistical analyses were performed using GraphPad Prism 10.0 software. Data normality was assessed with the Shapiro–Wilk test, and comparisons were made using one‐way or two‐way analysis of variance (ANOVA), followed by Tukey's or Šídák's post hoc test for multiple comparisons (*p* < 0.05).

## RESULTS

### Rheological analysis

**FIGURE 1:**
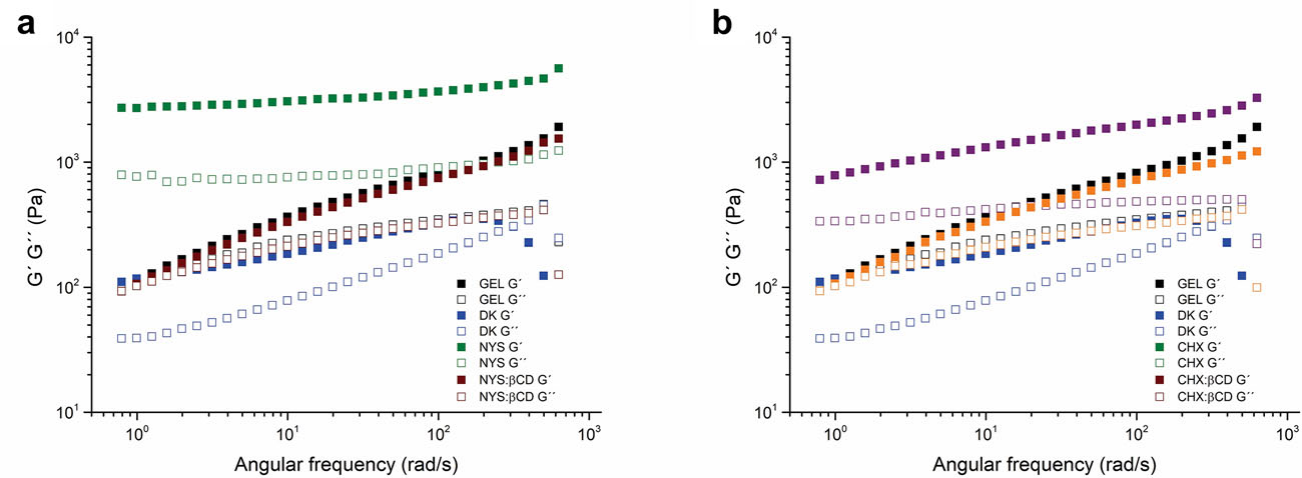
Representative graphs showing the elastic modulus (G′) and viscous modulus (G″) of the study groups at 37°C. (a): NYS; (b): CHX.

Figure 1 shows the viscoelastic parameters *G*′ (Pa) and *G*″ (Pa) obtained for the study groups. Rheological analysis showed that the viscoelastic properties of mucoadhesive formulations were frequency‐dependent, and the storage modulus (*G′*) was greater than the loss modulus (*G″*), indicating the prevalence of the elastic component over the viscous one (tan(δ) < 1; Table 1). All samples exhibited predominantly elastic behavior, characteristic of the gel state. However, noncomplexed groups showed higher values of *G′* and *G″* than complexed samples, which were similar to the GEL group.

**TABLE 1:** Viscoelastic parameters *G′*, *G″*, and tan(δ) at 1 and 10 Hz.

	1 Hz			10 Hz		
Samples	*G*′ (Pa)	*G*″ (Pa)	Tan(δ)	*G*′ (Pa)	*G*″ (Pa)	Tan(δ)
GEL	299	209	0.69	706	325	0.45
DK	167	66	0.39	293	157	0.53
NYS	2951	735	0.24	3495	864	0.24
NYS:βCD	272	189	0.69	644	302	0.47
CHX	1192	400	0.33	1850	474	0.25
CHX:βCD	273	186	0.67	630	289	0.45

### Mucoadhesive properties

Table 2 summarizes the results of the mucoadhesive properties of the formulations assessed in an in situ mucoadhesion test.

**TABLE 2:** Mean values ± standard deviations of the mucoadhesive properties of the formulations assessed in the mucoadhesion test using porcine mucosa.

Group	Adhesiveness (N)	Work of Adhesion (N.s)	Debonding Distance (mm)
GEL	0.57 ± 0.11 A	2.97 ± 0.34 A	17.62 ± 1.40 A
DK	0.51 ± 0.18 A	1.11 ± 0.07 B	4.08 ± 0.61 C
NYS	0.23 ± 0.07 A	1.58 ± 0.11 B	10.89 ± 2.62 B
NYS:βCD	0.44 ± 0.10 A	2.91 ± 0.17 A	17.76 ± 0.79 A
CHX	0.20 ± 0.14 A	1.22 ± 0.34 B	7.07 ± 1.47 C
CHX:βCD	0.40 ± 0.12 A	2.82 ± 0.37 A	17.60 ± 1.08 A

*Note*: Different letters indicate statistically significant differences between groups (one‐way ANOVA, Tukey's test; *p* < 0.05).

No statistically significant differences were observed between the groups regarding the adhesiveness of the formulations (*p* > 0.05). The highest work of adhesion and debonding distance were observed in the GEL and complexed groups (*p* < 0.05), with no significant differences between them (*p* > 0.05). DK and noncomplexed formulations exhibited inferior mucoadhesion properties. The lowest values were recorded in the DK, NYS, and CHX groups (*p* < 0.05).

### Antifungal potential

**FIGURE 2:**
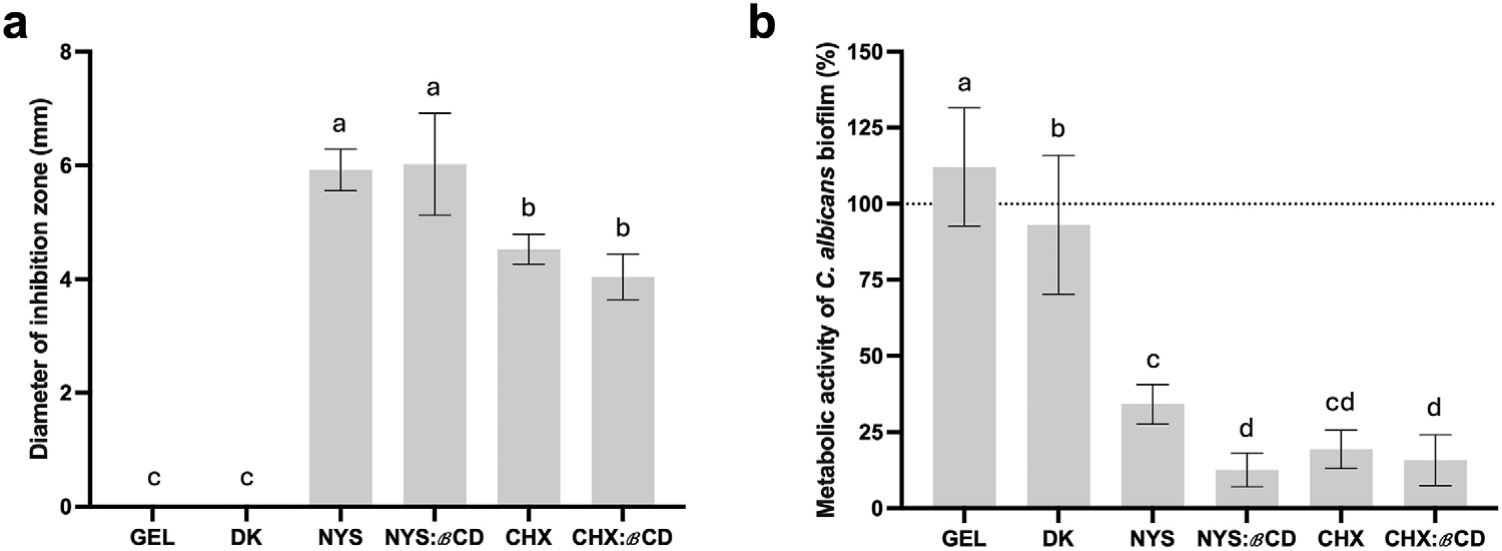
Graphs representing the antifungal potential of the tested formulations. (a) Ability to inhibit the growth of *C. albicans* in solid culture medium: inhibition zone (mm); (b) metabolic activity of the *C. albicans* biofilm (%). The dashed line indicates metabolic activity of the negative control group (biofilm treated without mucoadhesive leachates; 100% = 0.956 absorbance value). *Different letters indicate statistically significant differences between groups (one‐way ANOVA, Holm–Šídák test; *p* < 0.05).

Figure 2 presents the results for the antifungal potential of the formulations, including the ability to inhibit *C. albicans* growth in solid culture medium (zone of inhibition, in mm) and the metabolic activity of the *C. albicans* biofilm, expressed as a percentage.

No zones of inhibition were observed in the GEL and DK groups. In contrast, the NYS and NYS:βCD formulations produced zones of inhibition of approximately 6 mm in diameter, which were significantly larger than those exhibited by the CHX and CHX:βCD groups, of approximately 5 mm (*p* < 0.05).

Regarding the metabolic activity of the *C. albicans* biofilm, the GEL and DK groups showed high metabolic activity, with significant differences between them (*p* < 0.05). The NYS group showed metabolic activity below 50% (*p* < 0.05), followed by the CHX group, which did not show significant differences when compared to the formulations containing drugs complexed with βCD (NYS:βCD and CHX:βCD). These latter groups demonstrated the lowest levels of *C. albicans* metabolic activity.

The means and standard deviations of the percentages of inhibition in the growth of *C. albicans* for the experimental formulations are presented in Table 3.

**TABLE 3:** Mean ± standard deviation values for percentage inhibition values (%) against *C. albicans* biofilm formation by the broth microdilution method (MIC).

	Dilution					
Group	50%	25%	12.5%	6.25%	3.12%	1.56%
GEL	43 ± 7 Ba	42 ± 0 Ba	33 ± 7 Ba	18 ± 9 Bb	13 ± 2 Cb	16 ± 8 Cb
DK	39 ± 13 Ba	36 ± 4 Ba	35 ± 5 Ba	29 ± 8 Ba	38 ± 5 Ba	34 ± 5 Ba
NYS	87 ± 6 Aa	90 ± 8 Aa	86 ± 5 Aa	92 ± 1 Aa	94 ± 1 Aa	93 ± 0 Aa
NYS: βCD	95 ± 1 Aa	93 ± 0 Aa	94 ± 3 Aa	95 ± 3 Aa	95 ± 2 Aa	78 ± 6 Ab
CHX	96 ± 3 Aa	90 ± 3 Aa	83 ± 5 Aa	88 ± 5 Aa	92 ± 2 Aa	93 ± 2 Aa
CHX: βCD	94 ± 3 Aa	91 ± 4 Aa	90 ± 5 Aa	91 ± 0 Aa	94 ± 0 Aa	92 ± 9 Aa

*Note*: Uppercase letters indicate statistically significant differences between groups within the same dilution; lowercase letters indicate differences between dilutions within the same group (two‐way ANOVA, Tukey's test; *p* < 0.05). Hundred percent inhibition corresponds to an absorbance value of 0.057.

Table 3 shows that the GEL group showed a reduction in inhibition percentages between dilutions, ranging from 43% to 13%. Similarly, the DK group demonstrated inhibition percentages consistently below 50% for all dilutions tested, with values ranging from 39% to 29%. The groups with drug‐loaded mucoadhesive gel showed higher inhibition percentages, with the MIC_90_ dilution being 1.56% for the NYS, CHX, and CHX:βCD groups, and 3.12% for the NYS:βCD group.

The medians and first quartile values of CFU/mL for the various dilutions tested are presented in Table 4.

**TABLE 4:** Median ± first quartile values for the log_10_ CFU/mL values obtained with the study groups.

	Dilution					
Group	50%	25%	12.50%	6.25%	3.12%	1.56%
GEL	0.5 ± 0.25	3.5 ± 2	14 ± 10	39.5 ± 22.5	TNTC	TNTC
DK	70 ± 53.5	TNTC	TNTC	TNTC	TNTC	TNTC
NYS	0 ± 0	0 ± 0	0 ± 0	0 ± 0	0 ± 0	0 ± 0
NYS:βCD	0 ± 0	0 ± 0	0 ± 0	10.5 ± 5	TNTC	TNTC
CHX	0 ± 0	0 ± 0	0 ± 0	2.5 ± 1	5.5 ± 2.5	4.5 ± 2
CHX:βCD	0 ± 0	0 ± 0	0 ± 0	0 ± 0	0 ± 0	0 ± 0

Abbreviation: TNTC, too numerous to count.

According to Table 4, the GEL and DK groups did not exhibit complete fungal inhibition at any of the serial dilutions tested. In contrast, the NYS and CHX:βCD groups showed no growth of *C. albicans* colonies when the well contents were seeded, even at the lowest concentrations tested in the cytocompatibility assays, with 1.56% being considered the MFC for these groups. For the NYS:βCD and CHX groups, the MFC was determined to be 12.5%.

### Cytocompatibility tests in an organotypic model of the oral mucosa

**FIGURE 3:**
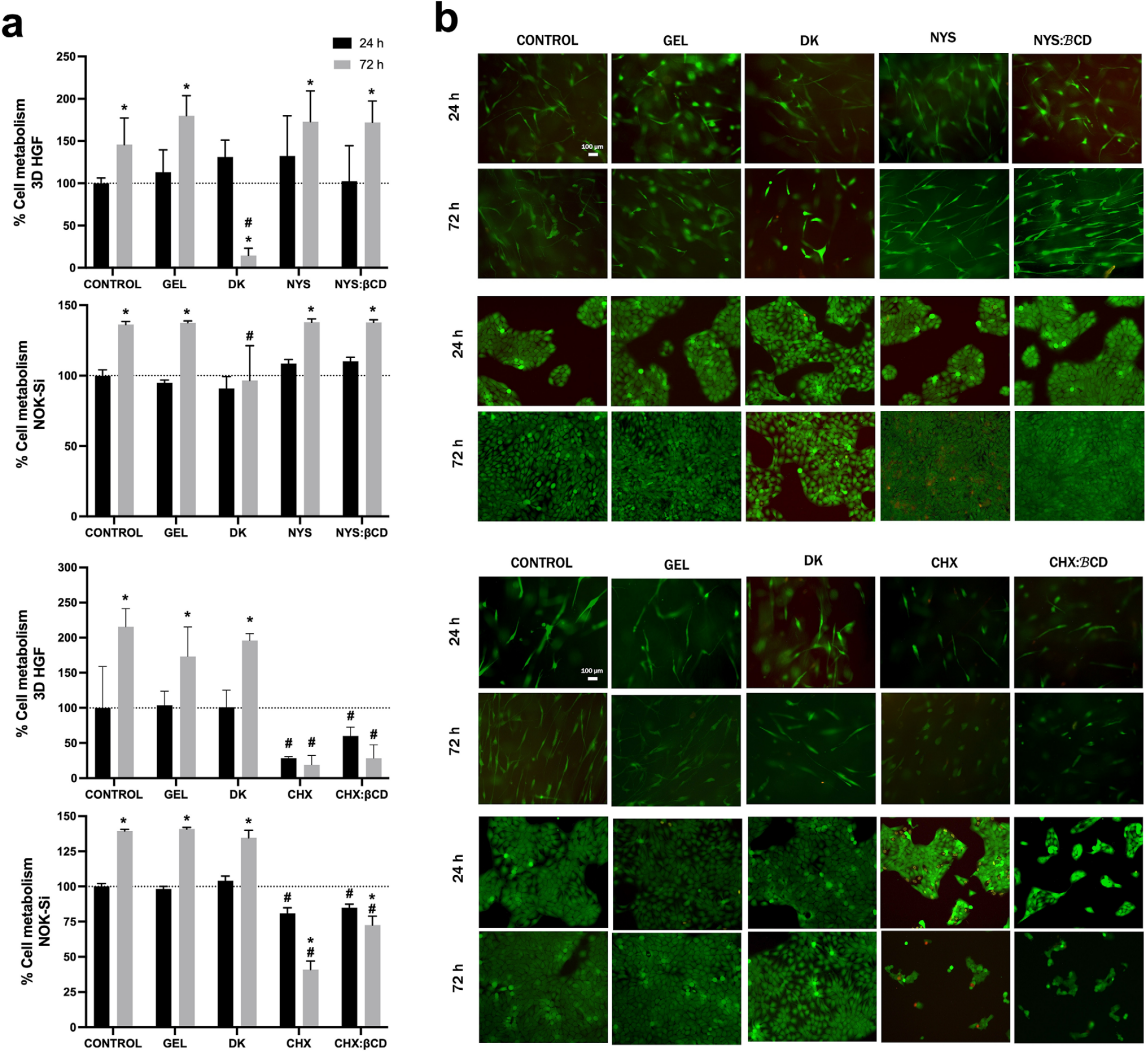
Cytocompatibility tests in an organotypic model of the oral mucosa. (a) Percentage of cellular metabolism (Alamar Blue assay) of human gingival fibroblast (HGF) in 3D culture or NOK‐Si in monoculture exposed to mucoadhesive leachates at concentrations of 12.5% (NYS groups) or 1.56% (CHX groups) (v/v) for 24 h or 72 h. Values were normalized to the negative control (exposed to culture medium only, without leachates for 24 h), set at 100% (dashed line). (b) Representative fluorescent images LIVE/DEAD cell viability assay for each group (live cells = green; dead cells = red). *Significantly different compared to the different time point, in the same study group. #Significantly different from negative control (two‐way ANOVA, Šídák test *p* < 0.05). Original magnification ×20. Scale bar 100 µm.

The results obtained with the quantitative Alamar Blue and qualitative LIVE/DEAD tests are presented in Figure 3.

In the comparative analysis with the NYS‐containing groups, both cells (HGF in 3D culture and NOK‐Si in monoculture) showed cell metabolism close to 100% at 24 h, with additional growth observed at 72 h (*p* < 0.05). No statistically significant difference was found between the GEL, NYS, and NYS:βCD groups compared to the negative control at any time (*p* > 0.05). In contrast, the DK group exhibited a statistically significant difference at 72 h (*p* < 0.05), showing lower cell metabolism in both cell cultures (*p* < 0.05). For HGF, the DK group demonstrated a significant decrease in cell metabolism after 72 h compared to the initial time point (*p* < 0.05). Representative images from the LIVE/DEAD assay supported the quantitative data, showing notable cell growth in HGF and NOK‐Si for all groups, except for the DK group (Figure 3).

In the CHX‐containing groups, all treatments exhibited lower cell metabolism at both 24 and 72 h compared to the negative control for both cell cultures (*p* < 0.05). No statistically significant difference was observed between the GEL and DK groups and the control (*p* > 0.05). The results of the Alamar Blue assay were consistent with the images of the LIVE/DEAD assay. Qualitatively, a noticeable reduction in the number of viable cells was observed in the CHX‐treated groups (Figure 3).

### Collagen synthesis potential

**FIGURE 4:**
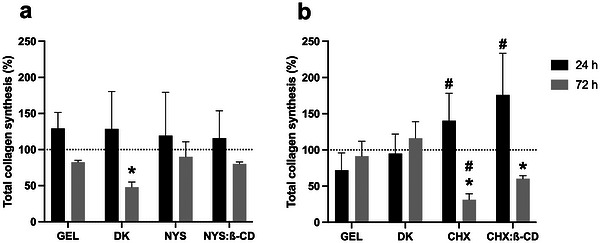
Percentage of total collagen synthesis in human gingival fibroblasts (HGFs) exposed for 24 and 72 h to leachates of mucoadhesives. (a) GEL, DK, NYS, and NYS:βCD 12.5% groups, and (b) GEL, DK, CHX, and CHX:βCD 1.56% groups. Values were normalized to the negative control (exposed to culture medium only, without leachates), set at 100% = 2405 absorbance value (dashed line). ^*^Significant difference compared to the different time points within the same experimental group. #Significant difference compared to the control (two‐way ANOVA, Šídák test; *p* < 0.05).

Figure 4 shows the total quantification of collagen synthesis (%) by HGFs when in contact with mucoadhesive leachates.

In the comparative analysis of the groups with NYS, no significant differences were observed when compared to the negative control. However, regarding the evaluation time, the DK group showed statistically significant differences after 72 h of evaluation (*p* < 0.0338).

In the groups treated with CHX, after 24 h, both CHX and CHX:βCD presented significantly higher percentages of collagen synthesis than the control (*p* < 0.05). In contrast, after 72 h of evaluation, the CHX group presented significantly lower collagen synthesis compared to the control (*p* < 0.05). When comparing the time points, a decrease in collagen synthesis was observed in the CHX (*p* = 0.0011) and CHX:βCD (*p* = 0.0006) groups after 72 h of exposure to the mucoadhesive leachates, when compared to the 24 h period.

## DISCUSSION

The present study evaluated a combined formulation strategy for the treatment of oral fungal infections using mucoadhesive gels containing NYS and CHX, both in their noncomplexed and βCD‐complexed forms. The incorporation of βCD contributed to improved antifungal performance at reduced drug concentrations, without adversely affecting the rheological or mucoadhesive properties of the gels. Importantly, the NYS‐containing formulations demonstrated cytocompatibility in an organotypic oral mucosa model and did not impair collagen production, providing further translational evidence of their suitability for therapeutic applications in mucosa. These findings expand on previous studies^13^, ^20^ by integrating advanced biological models to corroborate the safety and potential clinical relevance of βCD‐complexed antifungal mucoadhesive formulations.

The rheological properties of the formulations are fundamental to determining their behavior and performance.^35^ The viscoelastic properties of mucoadhesive gels were evaluated at 37°C to characterize them under intraoral conditions. All formulations presented predominantly elastic behavior, characterized by a well‐organized internal structure capable of resisting deformation and recovering its shape after stress. This viscoelastic profile contributes to the mechanical stability of the system, preventing phase separation or flow after application, which is crucial for ensuring prolonged contact with mucosal surfaces. Interestingly, the NYS:βCD and CHX:βCD formulations exhibited similar viscoelastic characteristics (*G*′ > *G″* and similar tan(δ) values) of the GEL sample, indicating that the incorporation of inclusion complexes did not alter the gelling nature of the preparations, suggesting that the internal structure of the system was not modified. Based on this behavior, it appears that the formulations were able to maintain their structural integrity and physical stability, without evidence of phase separation or degradation. On the other hand, the DK group and gels containing the noncomplexed drugs (in their raw form; NYS and CHX) exhibited *G′* and *G″* values, respectively, lower and higher than GEL and complexed samples. These performances are attributed to the greater solubility of the inclusion complexes compared to raw drugs, where the former disperses at a molecular level, whereas the latter exhibits a coarse dispersion.^36^

The rheological properties directly influenced the mucoadhesive performance of the formulations, as the DK, NYS, and CHX groups also demonstrated reduced mucoadhesive capacity. The NYS:βCD and CHX:βCD formulations exhibited significantly higher peel force and debonding distances compared to other groups. Adhesion work is a parameter related to the energy required to detach the formulation from the mucosa; that is, high values indicate greater adhesion. Similar mucoadhesion results between the complexed formulations and the GEL group corroborate the rheology analysis, indicating that these samples are physically stable with homogeneous drug incorporation. These findings suggest that the formulation developed with antifungal agents complexed with βCD exhibited desirable properties that align with the intended purpose. The incorporation of βCD enhanced the solubility and stability of the active ingredients,^13^ resulting in formulations with favorable rheological, texture, and mucoadhesive characteristics. These properties are beneficial for improving the therapeutic efficacy of antifungals when applied to mucosal surfaces, as they favor increased retention time and facilitate application.^37^

In this study, *C. albicans* SC5314 was selected because it is the most extensively used reference strain in biofilm studies of drug delivery systems from polymeric matrices,^10^, ^14^, ^21^, ^38^, ^39^, ^40^, ^41^ including those with mucoadhesive gels tested in the present investigation.^13^, ^20^ Additionally, it is the predominant etiological agent of oral and esophageal candidiasis, responsible for approximately 70%–80% of clinical cases and colonizing up to 90% of high‐risk patients, making it the most clinically representative model for initial antifungal evaluation.^42^ Recent evidence indicates that SC5314 harbors a rare dominant allele of the Rob1 transcription factor, associated with increased filamentation, biofilm formation, and persistence in the oral environment.^43^ The strain is therefore widely used as a reference isolate of *C. albicans*, based on its fully sequenced genome and its high clinical representativeness.^44^ When exposed to antifungal agents, SC5314 exhibits rapid loss of heterozygosity accompanied by marked genomic and phenotypic plasticity, manifested by morphological variability, chromosomal instability, and alterations in antifungal susceptibility.^44^ These characteristics indicate that SC5314 is genetically stable under basal conditions, but highly responsive to antifungal pressure, reflecting the adaptive capacity of *C. albicans* during antifungal therapy. Consequently, SC5314 can be considered a biologically robust and clinically significant model for evaluating antifungal efficacy and fungus–drug interactions in oral infections associated with biofilm. On the other hand, it is recognized that antifungal susceptibility can vary between clinical isolates and non‐*albicans* species,^45^, ^46^ including *Nakaseomyces glabratus*, which frequently exhibits reduced susceptibility and distinct behavior in biofilms. Therefore, future studies should incorporate multiple clinical strains and non‐*albicans* species to broaden the applicability of the findings.

In terms of antifungal activity, it is noteworthy that neither the GEL group nor the standard treatment with 2% miconazole gel (DK group) was effective against *C. albicans* in any of the assays performed. Although the GEL formulation showed no antifungal activity in this study, it is important to recognize that chitosan possesses inherent antimicrobial properties. Its activity, however, is highly dependent on factors such as molecular weight, degree of deacetylation, pH, and ionic strength.^47^ The low‐molecular‐weight chitosan (50,000–190,000 Da; 75%–85% deacetylation) used in the formulation may potentially exhibit antibacterial activity under acidic conditions, which could be evaluated in future studies aimed at better characterizing the antimicrobial spectrum of the mucoadhesive system.

The finding also corroborates the results of a randomized controlled clinical trial, in which miconazole gel 2% also failed to significantly reduce palatal inflammation or eradicate *Candida* from patients’ dentures and palates.^48^ In contrast, in this study, the groups containing NYS and CHX, both in noncomplexed and complexed forms, showed clear antifungal efficacy, with only minor differences between them. However, while the noncomplexed antifungal exhibited similar efficacy to the complex inclusion formulations, the complexed formulations required much lower drug concentrations to achieve the same results. Specifically, the amount of NYS needed was more than 50% lower, and the required concentration of CHX was more than six times lower compared to the formulations with the free drug. By reducing the required concentrations of active ingredients, complexation with βCD may help minimize the potential for systemic toxicity and adverse effects.^15^ These findings corroborate an animal model study, which showed that a modified soft relining material containing noncomplexed or βCD‐complexed antifungals promoted tissue recovery in palates with denture stomatitis, requiring much lower concentrations of the complexed antifungals.^41^

The combination of biological assays employed in this study produced robust findings regarding the antifungal performance of the mucoadhesive formulations. The halo inhibition assay was an initial screening tool to visually assess antifungal diffusion and inhibition zones. However, its interpretation must consider the diffusion limitations in solid media related to the viscosity, molecular weight, composition, and solubility of mucoadhesive formulations. Furthermore, it is an essentially qualitative or semi‐quantitative test that does not provide precise values of effective concentration, failing to distinguish between fungistatic and fungicidal effects.^49^ To overcome the limitations of the halo inhibition assay and provide stronger evidence of antifungal activity in biofilms and in vivo conditions, mucoadhesive formulations were also evaluated by the following assays: MIC, which is a quantitative, standardized, and comparable analysis across studies; MFC, to differentiate fungistatic and fungicidal activity, providing therapeutically relevant data for the formulations^50^; and XTT, to assess metabolic activity, which is useful for evaluating fungal biofilms and detecting sublethal effects, complementing MIC/MFC analysis by showing the physiological impact on the fungus.^51^

From a clinical perspective, the rheological behavior of the mucoadhesive gels contributes to their ability to spread over the mucosa and resist salivary washing, helping to maintain the formulation at the infection site, although mucoadhesion is primarily governed by polymer–mucin interactions. This retention is important for topical treatment of oral candidiasis. Additionally, the antifungal results were based on leachates from the formulation, which reflect the fraction of the drug effectively released rather than the total amount incorporated, offering a more clinically relevant measure of bioavailable drug levels.

In the cytocompatibility assays, the use of NOK‐Si keratinocytes and HGF fibroblasts in the organotypic model used in the present study aimed to reproduce the structure and function of the oral mucosa. Keratinocytes form the outermost epithelial layer, ensuring mucosal integrity, while fibroblasts contribute to the production of the extracellular matrix and tissue maintenance. The interaction between these cell types is necessary to replicate the physiological environment of the oral mucosa, as fibroblasts influence epithelial differentiation, proliferation, and response to external stimuli. This model provides a biologically relevant system to evaluate the cytocompatibility of mucoadhesives developed under conditions that resemble the in vivo environment.^52^, ^53^

Formulations containing NYS‐βCD showed minimal cytotoxicity at their MIC and MFC, with cell metabolism close to 100% in both HGF and NOK‐Si cultures after 24 and 72 h, indicating that they are biocompatible treatments. This contrasts with the formulations containing CHX, which showed a significant reduction in metabolic activity, particularly at 72 h, suggesting that CHX cytotoxicity may limit its use in long‐term applications. Zhang et al.^12^ demonstrated that CHX cytotoxicity is dose‐dependent, as the survival rate of HGF significantly reduced after 4 h exposure to a 1/512 dilution. Although it was hypothesized that complexing CHX with βCD would reduce the cytotoxicity of the drug, this expectation was not supported by the results. While the complexation allowed for the use of lower concentrations, optimizing the antifungal potential of CHX, it did not significantly reduce its cytotoxic effects. Taken together, these results indicate that the biological response to CHX constitutes an important limitation, highlighting the narrow therapeutic window of this agent when incorporated into delivery systems designed for mucosal contact. The data further suggest that additional improvements in CHX formulations will be necessary, including the exploration of different complexation ratios with βCD, modulation of release kinetics, or the use of combined approaches, before mucoadhesive gels containing this drug can be considered for clinical application.

This investigation provided relevant insights into the potential of these formulations for tissue repair and regeneration through the evaluation of collagen synthesis. CHX may initially stimulate collagen synthesis, but prolonged exposure can lead to reduced collagen production, likely due to its cytotoxic effects. The results observed in the NYS‐containing groups further support the notion that βCD complexation helps mitigate the adverse impact of potentially cytotoxic agents on tissue regeneration. These results are in agreement with studies that have recommended βCD‐based formulations for tissue regeneration.^54^, ^55^

## CONCLUSION

The results of the present study support the relevance of complexation with βCD as a complementary strategy to improve the performance of mucoadhesive antifungal formulations, particularly by allowing effective antifungal activity at reduced drug concentrations, while preserving the physicochemical properties of the gels. The use of an organotypic oral mucosa model and the assessment of collagen synthesis provide additional translational evidence on the biological safety and tissue compatibility of these systems. These aspects are not commonly addressed in studies of antifungal formulations. In this context, NYS‐based formulations demonstrated greater biocompatibility and translational potential, while gels containing CHX highlighted important limitations related to biological tolerance due to the high cytotoxicity presented, regardless of complexation with βCD, emphasizing the need for further optimization prior to clinical use.

Overall, these findings reinforce the potential of βCD‐based mucoadhesive systems as locally applied antifungal therapies for oral mucosal infections, particularly when formulation development prioritizes drug retention, cytocompatibility, and tissue response. The ability to maintain antifungal efficacy using lower drug loads should be viewed as a strategic enhancement of topical antifungal administration, not a paradigm shift, contributing incremental, yet clinically relevant, evidence to the field.

## CONFLICT OF INTEREST STATEMENT

The authors declare no conflicts of interest.

## Data Availability

Data will be made available on request.
